# Familial childhood-onset progressive cerebellar syndrome associated with the *ATP1A3* mutation

**DOI:** 10.1212/NXG.0000000000000145

**Published:** 2017-03-27

**Authors:** Fatima Jaffer, Katherine Fawcett, David Sims, Andreas Heger, Henry Houlden, Michael G. Hanna, Helen Kingston, Sanjay M. Sisodiya

**Affiliations:** From the Department of Neurosciences (F.J.), King's College Hospital, London; MRC Centre for Neuromuscular Diseases and Department of Molecular Neuroscience (F.J., H.H., M.G.H.), UCL Institute of Neurology; MRC Computational Genomics Analysis and Training Programme (K.F., D.S., A.H.), University of Oxford; Central Manchester University Hospitals (H.K.); and NIHR UCLH Biomedical Research Centre (S.M.S.), Department of Clinical and Experimental Epilepsy, UCL Institute of Neurology, Queen Square, UK.

The allelic disorders rapid-onset dystonia-parkinsonism (RDP), alternating hemiplegia of childhood (AHC), and CAPOS/CAOS syndrome (cerebellar ataxia, areflexia, pes cavus, optic atrophy, and sensorineural deafness) are caused by *ATP1A3* mutations.^1–3^ Intermediate RDP-AHC phenotypes are emerging. Positional mutations 274, 583, 867, and 923 lead to both RDP and AHC, suggesting different pathomechanisms.^4,5^ The E818K mutation underlies all reported cases of CAPOS/CAOS, including an AHC-CAPOS overlap syndrome.^6^ We report a family with features of all 3 *ATP1A3*-spectrum disorders.

## Case report.

The proband (II-3; figure, A) is 49 years old, born at 32 weeks of gestation with pre-eclampsia complicating pregnancy. Development was delayed, walking at 28 months with progressive gait ataxia, and speaking at 30 months. Hypertonia and choreoathetosis were present at this time. Aged 5 years, she developed fever-induced acute-onset generalized hypotonia and dysarthria. Motor weakness persisted for 8 months, requiring neurorehabilitation to reacquire independent ambulation. Aged 6 years, she developed limb incoordination and dysarthria, worsening during febrile illnesses. Childhood-onset painful limb spasms and stimulus-sensitive finger myoclonus were reported. At 30 years, she noted further worsening of dysarthria and ataxia. Examination at 49 years revealed short stature, low-set ears, scoliosis, and pes planus. She had marked cerebellar signs with dysarthria, dysmetria, square wave jerks, intention tremor, and gait ataxia and spasticity with preserved muscle strength, choreoathetosis, finger myoclonus, and cervical dystonia. Reflexes were absent and sensation intact. Fundoscopy and hearing were normal. MRI showed cerebellar atrophy.

**Figure:**
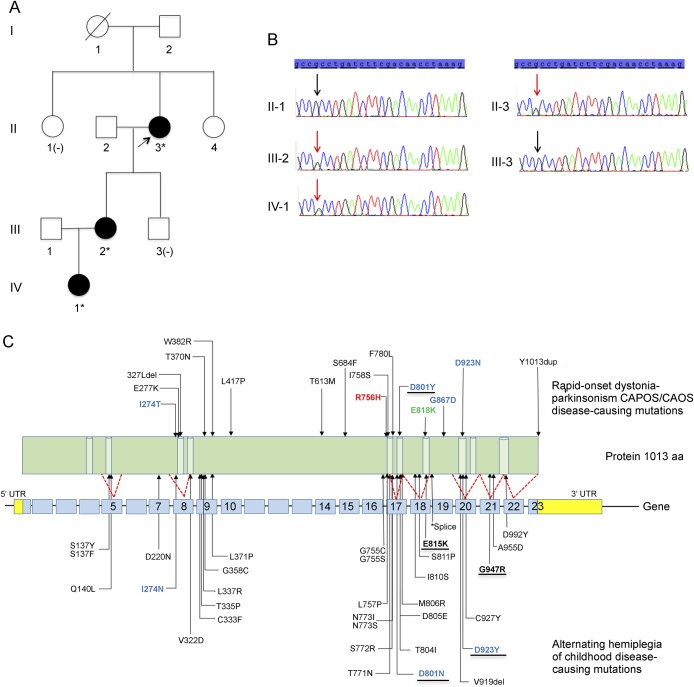
Pedigree and genetics (A) Family pedigree: no clinical information is available on the parents of the proband; Sanger sequencing confirmed the presence of c.2267G>A; p.Arg756His (indicated by *) in the *ATP1A3* gene in all affected family members and was absent in unaffected family members, II-1 and III-3 (indicated by [-]). (B) Validation of mutation by Sanger sequencing. Chromatograms showing segregation of the variant with disease: present in affected family members II-3, III-2, and IV-1 and absent in II-1 and III-3. (C) Mutation map of the *ATP1A3* gene. *ATP1A3* mutation map showing all published mutations associated with rapid-onset dystonia-parkinsonism (RDP) (top panel), CAPOS/CAOS (top panel; highlighted in green), and alternating hemiplegia of childhood (AHC) (bottom panel). Mutations underlined indicate frequently reported variants associated with the disease; mutations in our patients are indicated in red; amino acid positions 274, 583, 923, and 801 highlighted in blue are mutated in both RDP and AHC; * indicates intronic splice-site variant c.2542 +1G>A.

Individual III-2 was delivered at term and had delayed development, sitting at age 10 months, speaking at 18 months, and walking at 27 months with ataxia. At 30 months, she transiently lost the ability to walk, with upper limb ataxia, lasting 3 weeks after a febrile illness. She had another episode after Varicella zoster infection, with acute-onset generalized hypotonia, dysarthria, and dysphagia lasting for 1 month, followed by slow recovery. Examination at age 5.5 years showed persistent cerebellar signs with dysarthria, gait, and limb ataxia. She continues to have episodic cerebellar deterioration during intercurrent illness, with a recovery period of 1–2 days and complete return to baseline function. Examination at 20 years showed dysarthria, dysmetria, square wave jerks, gait ataxia, spasticity with normal muscle strength, global hyporeflexia with down-going plantar responses, and normal sensation. Athetoid movement of the hands, finger myoclonus, and scoliosis were present. There was no visual or hearing impairment. Brain MRI shows nonspecific mild periventricular white-matter changes and normal cerebellar volume.

Individual IV-1 presented at age 8 months with generalized weakness during a febrile illness, taking 3 weeks to recover and be able to sit independently again. She started walking at age 17 months, and at 2 years, was ataxic and unable to walk independently. Vision and hearing were normal.

Mutation analysis for spinocerebellar ataxias 1, 2, 3, 5, 7, and 17, dentatorubral-pallidoluysian atrophy, ferritin light chains, and common mitochondrial DNA mutations in the proband were negative, as were plasma acylcarnitine profile, lactate, ammonia, and amino acids. Whole-exome sequencing revealed a deleterious missense mutation, c.2267G>A; p.Arg756His (R756H), in *ATP1A3*, confirmed by Sanger sequencing and shown to segregate with the disease (figure, B).

## Discussion.

Infantile-onset RDP with motor delay and initial fluctuations evolving into persistent dystonia and ataxia is reported with R756H mutations.^7^ With the exception of IV-1, our patients presented with motor delay prior to paroxysmal motor symptoms, with a protracted recovery period, consistent with this picture. The development of a progressive cerebellar syndrome with superimposed fever-induced fluctuation is a key clinical feature in all three affected members of our family as is global hyporeflexia, suggesting some features also of CAPOS/CAOS syndrome, without visual or auditory involvement. Development of gait ataxia preceding episodic features broadens the phenotype away from CAPOS/CAOS and infantile-onset RDP.

CAPOS are associated with pes cavus: recently, cases without are reported.^3^ Our proband has flat feet, and she and her daughter have scoliosis, extending the musculoskeletal phenotype of *ATP1A3*-spectrum syndromes. The age at onset in this family fits with CAPOS/CAOS and AHC, and the development of pyramidal signs and an extrapyramidal disorder observed is more in keeping with nonparoxysmal AHC/RDP features. Overall, the phenotypic presentation has features of all three *ATP1A3*-spectrum disorders. The early-onset cerebellar ataxia prior to paroxysmal symptoms and extended musculoskeletal involvement is novel. Early MRI is unavailable: however, cerebellar atrophy in adulthood is observed in the proband, suggesting possible progression and development of fixed deficits observed in other neurologic channelopathies.

Reporting unique *ATP1A3*-spectrum phenotypes facilitates genotype-phenotype correlation, although some phenotypic aspects may escape detection, directs functional studies and possible therapeutic targets. We propose that *ATP1A3* mutation analysis is considered in patients with delayed motor development and childhood- or early-onset progressive cerebellar syndromes with superimposed acute motor fluctuations.

## References

[1] Rosewich H, Ohlenbusch A, Huppke P, et al The expanding clinical and genetic spectrum of ATP1A3-related disorders. Neurology 2014;82:945–955. 24523486 10.1212/WNL.0000000000000212

[2] Demos MK, Van Karnebeek CDM, Ross CJD, et al A novel current mutation in ATP1A3 causes CAPOS syndrome. Orphanet J Rare Dis 2014;9:15. 24468074 10.1186/1750-1172-9-15 PMC3937150

[3] Heimer G, Sadaka Y, Israelian L, et al CAOS-episodic cerebellar ataxia, areflexia, optic atrophy, and sensorineural hearing loss: a third allelic disorder of the ATP1A3 gene. J Child Neurol 2015;30:1749–1756. 25895915 10.1177/0883073815579708

[4] Sasaki M, Ishii A, Saito Y, Hirose S Intermediate form between alternating hemiplegia of childhood and rapid-onset dystonia-parkinsonism. Mov Disord 2014;29:153–154. 24123283 10.1002/mds.25659

[5] Nicita F, Travaglini L, Sabatini S, et al Childhood-onset ATP1A3-related conditions: report of two new cases of phenotypic spectrum. Parkinsonism Relat Disord 2016;30:81–82. 27268479 10.1016/j.parkreldis.2016.05.029

[6] Rosewich H, Weise D, Ohlenbusch A, Gärtner J, Brockmann K Phenotypic overlap of alternating hemiplegia of childhood and CAPOS syndrome. Neurology 2014;83:861–863. 25056583 10.1212/WNL.0000000000000735

[7] Brashear A, Mink JW, Hill DF, et al ATP1A3 mutations in infants: a new rapid-onset dystonia-Parkinsonism phenotype characterized by motor delay and ataxia. Dev Med Child Neurol 2012;54:1065–1067. 22924536 10.1111/j.1469-8749.2012.04421.x PMC3465467

